# Modified dendritic cell-derived exosomes activate both NK cells and T cells through the NKG2D/NKG2D-L pathway to kill CML cells with or without T315I mutation

**DOI:** 10.1186/s40164-022-00289-8

**Published:** 2022-06-07

**Authors:** Zhuanyun Du, Zhenglan Huang, Xi Chen, Guoyun Jiang, Yuhang Peng, Wenli Feng, Ningshu Huang

**Affiliations:** 1 Department of Clinical Hematology, Key Laboratory of Laboratory Medical Diagnostics Designated By Ministry of Education, School of Laboratory Medicine, Chongqing Medical University, No.1, Yixueyuan Road, Yuzhong District, Chongqing, 400016 China; 2 Center for Clinical Molecular Medicine, Ministry of Education Key Laboratory of Child Development and Disorders, National Clinical Research Center for Child Health and Disorders, China International Science and Technology Cooperation Base of Child Development and Critical Disorders, Children’s Hospital of Chongqing Medical University, Chongqing, China; 3 Department of Clinical Laboratory, Chongqing Key Laboratory of Pediatrics, Ministry of Education Key Laboratory of Child Development and Disorders, National Clinical Research Center for Child Health and Disorders, China International Science and Technology Cooperation Base of Child Development and Critical Disorders, Children’s Hospital of Chongqing Medical University, Chongqing, China

**Keywords:** Chronic myeloid leukemia, Dendritic cell-derived exosomes, RAE-1γ, T cells, NK cells

## Abstract

**Background:**

Tyrosine kinase inhibitors have achieved quite spectacular advances in the treatment of chronic myeloid leukemia (CML), but disease progression and drug resistance that related to the T315I mutation, remain major obstacles. Dendritic cell-derived exosomes (Dex) induce NK cell immunity, but have yet to achieve satisfactory clinical efficacy. An approach to potentiate antitumor immunity by inducing both NK- and T-cell activation is urgently needed. Retinoic acid early inducible-1γ (RAE-1γ), a major ligand of natural killer group 2 member D (NKG2D), plays an important role in NK-cell and T-lymphocyte responses. We generated RAE-1γ enriched CML-specific Dex (CML-RAE-1γ-Dex) from dendritic cells (DCs) pulsed with lysates of RAE-1γ-expressing CML cells or T315I-mutant CML cells, aiming to simultaneously activate NK cells and T lymphocytes.

**Methods:**

We generated novel CML-RAE-1γ-Dex vaccines, which expressed RAE-1γ, and were loaded with CML tumor cell lysates. NK cells or T lymphocytes were coincubated with CML-RAE-1γ-Dex vaccines. Flow cytometry was performed to evaluate the activation and proliferation of these immune cells. Cytokine production and cytotoxicity toward CML cells with or without the T315I mutation were detected by ELISPOT, ELISA and LDH assays. CML models induced by BCR-ABL or BCR-ABL^T315I^ were used to determine the immunological function of Dex in vivo.

**Results:**

Herein, CML-RAE-1γ-Dex were prepared. CML-RAE-1γ-Dex effectively enhanced the proliferation and effector functions of NK cells, CD4^+^ T cells and CD8^+^ T cells, which in turn produced strong anti-CML efficacy in vitro. Moreover, CML-RAE-1γ-Dex-based immunotherapy inhibited leukemogenesis and generated durable immunological memory in CML mouse models. Similar immune responses were also observed with imatinib-resistant CML cells carrying the T315I mutation.

**Conclusions:**

This approach based on CML-RAE-1γ-Dex vaccines may be a promising strategy for CML treatment, especially for cases with the T315I mutation.

**Supplementary Information:**

The online version contains supplementary material available at 10.1186/s40164-022-00289-8.

## Background

Chronic myeloid leukemia (CML) is a myeloproliferative disorder involving the hematopoietic stem cell compartment that is hallmarked by a typical reciprocal translocation between chromosomes 9 and 22. The formed bcr-abl fusion gene is translated into the BCR-ABL oncoprotein which displays constitutive tyrosine kinase activity and results in CML occurrence and progression [1]. Imatinib, a representative tyrosine kinase inhibitor (TKI), improves the life expectancy of CML patients [2]. However, over 20% of patients cannot achieve disease eradication or overcome drug resistance or intolerance [3]. Point mutations are mostly responsible for resistance to TKIs. Unlike other mutations, the “gatekeeper” mutation T315I not only is the most commonly identified mutation, but also is associated with a poor response to newly developed TKIs [4]. Ponatinib, a third generation TKI exhibits a great effect but notable toxicities [5]. Thus, exploration of novel therapeutic strategies to improve the current immunotherapeutic status of CML patients or patients with the T315I mutation is urgently needed.

Cancer immunotherapy has been found to be a powerful therapeutic modality for targeting malignant tumor cells and has revolutionized hematological malignancy therapy [6–8]. In CML, oncoproteins, whether BCR-ABL or BCR-ABL^T315I^, are necessary for tumor cell survival. CML cells cannot escape immunotherapy by decreasing oncoprotein expression. Therefore, CML has unique antigens or drug resistance-related antigens that can be specifically targeted with immunotherapy [9, 10]. However, it is difficult for traditional immunotherapies including adoptive cell transfer or monoclonal antibody (mAb) therapies to recognize these oncoproteins because they are located in the cytoplasm rather than on the cell membrane [9]. Dendritic cells (DCs) as potent antigen-presenting cells (APCs), can internalize tumor lysates or antigens and induce antitumor responses by CD8^+^ cytotoxic T lymphocyte (CTLs) and CD4^+^ T helper (Th) cells [11]. Regrettably, the objective clinical immune response rates achieved with DC-based vaccines are less than 15% [12, 13]. Immunosuppression occurring in the tumor microenvironment or T-cell dysfunction in CML patients impairs the immune activity of DCs [14, 15]. Consequently, it is difficult to cure CML patients with DC-based therapeutic vaccines. Dendritic cell-derived exosomes (Dex) vaccines with better bioavailability and biostability are superior to DCs in resistance to tumor-related immunosuppression and thus have superior advantages in achieving antitumor efficacy [16]. Notably, the application of Dex vaccines in vivo is safe and well-tolerated [17–19]. Nevertheless, the application of CML-specific Dex for CML treatment has not been reported.

Dex are released by DCs via the classic exosome generation pathway, in which multivesicular endosomes (MVEs) fuse with the plasma membrane [20, 21]. Additionally, Dex carry functional immune-stimulatory components involved in antigen presentation, including major histocompatibility complex class I and II (MHC I/MHC II) on their surface [22–24]. Due to Dex’s retaining antigen-presenting ability of the parental DCs, they exhibit expression of MHC I- and MHC II-peptide complexes for priming antigen-specific CD8^+^ and CD4^+^ T cells [25, 26]. However, despite inducing natural killer (NK)-cell activation and proliferation, Dex have rarely elicited tumor antigen-specific T-cell functions in Dex-based clinical trials. Hence, only limited clinical efficacy has been observed [17–19, 27]. These results indicate that in the clinic, it is difficult to suppress tumors merely via the antitumor effect of NK cells induced by Dex. Boosting antitumor immunity by activating multiple immune cells including NK cells and T lymphocytes is required. Thus, modifying Dex to induce a T-cell response and simultaneously improve NK-cell activation, which would enhance the innate and adaptive antitumor efficacy of Dex vaccines, is a potential strategy to eradicate tumor cells. Another possible reason for treatment failure in Dex-based clinical trials is the use of MHC-restricted peptides [17–19]. These peptides are suitable for use in only some patients and often are ineffective due to the frequent mutations in tumors [28]. Conversely, allogeneic tumor cell lysates, could supply a wider variety of tumor antigens and elicit a strong immune response as effective antigen sources [29]. The application of tumor cell lysates is more conducive to personalized treatment even if a patient’s tumor mutates. Therefore, CML-specific Dex may be generated to overcome imatinib resistance by loading DCs with lysates of CML cells with the T315I mutation.

Recently, extensive research has demonstrated that the expression of natural killer group 2 member D (NKG2D) ligands (NKG2D-L) in Dex, can help to promote the activation of NK cells by directly interacting with the receptor NKG2D [27]. As a key activating immune receptor, NKG2D is expressed on nearly all NK cells, most natural killer T (NKT) cells, γδ T cells, CD8^+^ T cells, and certain CD4^+^ T cells [30, 31]. Moreover, studies have indicated that the NKG2D/NKG2D-L pathway provides costimulatory signals to T lymphocytes [32, 33]. Based on current findings, as a major murine NKG2D-L for NKG2D, retinoic acid early inducible-1γ (RAE-1γ) can bind to NKG2D which plays a prominent function in mediating NK-cell and T-cell activation [34, 35]. Given that current Dex vaccines have been observed to have limited clinical effects and minor antigen-specific T-cell function in clinical trials, we proposed to generate RAE-1γ-expressing CML-specific Dex by pulsing DCs with RAE-1γ-enriched CML cell lysates; these Dex in turn will prime T cells and NK cells to produce strong anti-CML efficacy via the NKG2D pathway. We also designed CML-specific Dex with RAE-1γ expression from DCs loaded with lysates of RAE-1γ-expressing CML cells with the T315I mutation to activate immune cells, with the goal of addressing imatinib resistance.

In this study, we generated RAE-1γ-enriched CML-specific Dex (CML-RAE-1γ-Dex) from murine DCs loaded with lysates of RAE-1γ-expressing CML cells. We showed that CML-RAE-1γ-Dex vaccines were able to simultaneously activate T and NK cells via the NKG2D/NKG2D-L pathway, leading to robust anti-CML effects in vitro and in vivo. In addition, we showed that RAE-1γ-enriched Dex loaded with lysates from T315I-mutant CML cells could also enhance the cytotoxic function of NK cells and T cells against CML cells with the T315I mutation. Herein, we may open up new avenues for Dex vaccines as an anti-CML immunotherapy.

## Materials and methods

### Lentiviral transduction of CML cells

Murine hematopoietic cells, BaF3 cells, were purchased from the Cell Culture Center, Institute of Basic Medical Sciences Chinese Academy of Medical Sciences and maintained in RPMI 1640 medium (Gibco, NY, USA) supplemented with 10% fetal bovine serum (FBS; Gibco, USA) and 1 ng/ml IL-3 (PeproTech, NJ, USA). The CML cell line BP210 and imatinib-resistant CML cell line BP210-T315I were derived from BaF3 cells transfected with P210 BCR-ABL-expressing retrovirus and P210 BCR-ABL^T315I^-expressing retroviruses, respectively [36, 37]. BP210 cells and BP210-T315I cells stably expressing BCR-ABL were derived by limited dilution cloning and were IL-3-independent; these cells were cultured in complete RPMI 1640 medium (RPMI 1640 medium supplemented with 10% FBS). BP210-mock, BP210-RAE-1γ, BP210-T315I-mock and BP210-T315I-RAE-1γ cells were established by transfecting BP210 cells or BP210-T315I cells with the corresponding lentivirus generated with the control CV186 vector or RAE-1γ-expressing CV186 vector. Seventy-two hours later, the transfectants were screened in fresh complete RPMI 1640 medium supplemented with puromycin (2 μg/ml, Solarbio, Beijing, China). Cells were stained with a PE-conjugated anti-RAE-1γ antibody (eBioscience, CA, USA) or PE-conjugated rat IgG2b kappa antibody (eBioscience, USA) and then subjected to flow cytometric detection.

### Preparation of tumor cell lysates

As established leukemia cell lines, BP210-mock, BP210-RAE-1γ, BP210-T315I-mock and BP210-T315I-RAE-1γ cells were collected and resuspended in phosphate-buffered saline (PBS) after being washed. Cells frozen instantly in liquid nitrogen were thawed in a 37 °C constant temperature water bath. Tumor lysates generated with three repeated freeze/thaw cycles were isolated from the cellular debris by centrifugation. The concentration of the supernatant was estimated with a BCA Protein Assay Kit (Biosharp, Anhui, China) after filtering through 0.22-μm filters (Millipore, Billerica, Massa-chusetts, USA), and the samples were stored at − 80 °C until use.

### Generation of DCs

Bone marrow cells were isolated by flushing the bone marrow cavity of tibias and femurs from 6-to 8-week-old male Balb/c mice purchased from the Laboratory Animal Center of Chongqing Medical University. Bone marrow-derived DCs (BMDCs) were obtained by inducing bone marrow cells in RPMI 1640 medium containing 10% FBS, 10 ng/ml granulocyte–macrophage colony-stimulating factor (GM-CSF) (Sino Biological, Beijing, China) and 5 ng/ml IL-4 (Sino Biological, China) [38]. On Day 7, DCs were mixed with tumor cell lysates (100 μg/ml) and cultured for 8 h. After the incubation with the lysates, the DCs were fed fresh complete RPMI 1640 medium containing 10 ng/ml GM-CSF and 5 ng/ml IL-4 with tumor necrosis factor-alpha (TNF-α) (10 ng/ml, Sino Biological) added to induce maturation. Immunophenotyping of freshly isolated DCs and mature DCs was performed by flow cytometry (Becton Dickinson, USA) as previously described [38]. A fluoroscein isothiocyanate (FITC)-conjugated anti-CD80 monoclonal antibody (mAb) (eBioscience, USA) was added to detect CD80 expression.

### Exosome isolation and purification

DCs were cultured in exosome-free medium; the exosomes were removed by ultracentrifugation at 100,000 g overnight. The supernatants of the DCs were isolated for exosome collection by centrifugation at 300 × g for 10 min to deplete cells and cellular fragments. Successive centrifugation steps were conducted to further purify the exosomes at 4 °C: 2,000 × g for 10 min and 10,000 × g for 30 min to remove debris. Afterward, the supernatant was filtered directly through a 0.22-μm membrane and ultracentrifuged at 100,000 × g for 70 min. Then, the precipitate was resuspended in precooled PBS, closely followed by ultracentrifugation for another 70 min at 100,000 g and 4 °C. The exosomes were stored for direct detection or kept at − 80 °C.

Dex indicate exosomes isolated from DCs induced to mature with TNF-α. BP210-mock-Dex (B-m-Dex) and BP210-T315I-mock-Dex (BT-m-Dex) represent exosomes that were released by mature DCs pulsed with whole tumor lysates of BP210-mock and BP210-T315I-mock cells, respectively. BP210-RAE-1γ-Dex (B-R-Dex) and BP210-T315I-RAE-1γ-Dex (BT-R-Dex) indicate exosomes derived from mature DCs loaded with whole tumor lysates of BP210-RAE-1γ and BP210-T315I-RAE-1γ cells, respectively.

### Exosome characterization

Exosomes suspended in PBS were added to Formvar and carbon-coated copper grids and then negatively stained with 2% uranyl acetate. Ultimately, exosomal morphology was imaged by transmission electron microscopy (TEM, Tecnai G2 Spirit, Holland) at a voltage of 80 kV. The concentration and size distribution of exosomes in suspension were measured by nanoparticles tracking analysis (NTA) with a ZetaView (ZetaView PMX 110, Germany).

### Western blot analysis

Western blotting was conducted according to a previously reported protocol [36]. Total protein from lysed whole DCs and exosomal preparations was separated by 10% SDS–PAGE. Primary antibodies targeting HRS (Santa Cruz, Delaware, CA, USA), Alix and tumor susceptibility gene 101 (TSG101) (Bimake, Houston, TX, USA) were purchased and used to identify exosomal markers at a dilution of 1:1000. A mAb specific for cytochrome C (Bimake, USA) and a RAE-1 pan specific polyclonal antibody (R&D Systems, Minneapolis, MN, USA) were used at dilutions of 1:1000 and 1:500, respectively.

### Exosome binding assay

To detect the efficiency of exosomal uptake by NK cells, Dex were labeled with the PKH26 dye from the PKH26 Red Fluorescent Cell Linker Kit (Sigma–Aldrich, St. Louis, MO, USA) following the manufacturer’s instructions. The exosomes were collected after being washed with PBS by ultracentrifugation. NK cells incubated with labeled exosomes were separately collected at 0 h and 6 h. The nuclei of the NK cells were stained with DAPI (Beyotime, Shanghai, China). The localization of exosomes and NK cells was observed and imaged by confocal fluorescence microscopy.

### T-cell and NK-cell isolation

T lymphocytes were isolated from the spleen of Balb/c mice (aged 6 to 8 weeks) via density centrifugation using Ficoll lymphocyte separation solution (TBD, Tianji, China). NK cells were isolated via centrifugation using a splenic NK-cell separation solution kit (TBD, China). NK cells were enriched to a higher purity from a fresh splenic single-cell suspension by positive selection with CD49b (DX5) MicroBeads (Miltenyi Biotec, Germany). The NK cells were then maintained in complete RPMI 1640 medium supplemented with 2 mM L-glutamine (Solarbio, China), 50 μmol/L β-mercaptoethanol (Sigma–Aldrich, USA) and 300 U/ml IL-2 (PeproTech, USA). Mouse T lymphocytes were cultured as previously reported [38].

### Flow cytometric analysis

NK cells (CD3^−^DX5^+^) were labeled with FITC-conjugated anti-CD3ε (clone: 145-2C11, eBioscience) and PE-Cyanine7-conjugated anti-CD49b (clone: DX5, eBioscience) and subsequently analyzed by flow cytometry. For CD4^+^ or CD8^+^ T-cell detection, cells were stained with FITC-conjugated anti-CD3ε and PE-Cyanine7-conjugated anti-mouse CD4 (clone: RM4-5, eBioscience) or a PerCP-Cyanine5.5-conjugated anti-mouse CD8α mAb (clone: 53–6.7, eBioscience). For immune cell activation, NK cells or T cells were stimulated with PBS or exosomes for 6 h and 36 h, respectively, and then the cells were stained with fluorochrome-labeled antibodies for 30 min protected from light. The cells were resuspended and then detected on a BD FACSCanto flow cytometer (BD Biosciences, USA). The antibodies used were as follows: APC-conjugated anti-CD69 (clone: H1.2F3, eBioscience), eFluor 450-conjugated anti-CD107a (LAMP-1, clone: eBio1D4B(1D4B), eBioscience), APC-conjugated anti-CD137 (4-1BB, clone: 17B5, eBioscience), and isotype control antibodies directly conjugated to the appropriate fluorescent dye (eBioscience, USA).

To investigate the levels of cytolytic mediators, NK cells or T lymphocytes were treated with PBS or relevant exosomes. Brefeldin A (eBioscience, USA) was added and then incubated for 4 h before cell collection. The samples were stained to identify characteristic cell-surface markers of immunocytes and then fixed with fixation buffer, followed by permeabilization (Invitrogen, USA). The cells were intracellularly labeled with APC-conjugated anti-perforin (clone: eBioOMAK-D, eBioscience), PE-conjugated anti-granzyme B (clone: NGZB, eBioscience) or isotype control antibodies (all eBioscience, USA) and evaluated by flow cytometry.

For blockade of NKG2D signaling, immune cells were preincubated with a blocking anti-mouse NKG2D mAb (10 μg/ml, R&D Systems, USA) for 30 min and then stimulated with exosomes. Rat IgG purchased from R&D Systems was used as a control (ctrl IgG). Then the incubated cells were collected, washed with PBS, and stained according to the experimental procedures described above.

### NK-cell and T-cell proliferation in vitro

Carboxyfluoresceine succinimidyl ester (CFSE; 2.5 μM, Invitrogen, USA) was added to freshly isolated NK cells or T lymphocytes and mixed gently and adequately protected from light. The cells were washed, then plated in 24-well plates, and separately cocultured with various types of exosomes for 72 h. For NK cells, the harvested cells were coincubated with a PE-conjugated anti-CD3ε antibody (clone: 145-2C11, Thermo Fisher, USA) and PE-Cyanine7-conjugated anti-DX5 antibody (eBioscience, USA) for 30 min. To identify CD4^+^ and CD8^+^ T cells, a PE-conjugated anti-CD3ε antibody (Thermo Fisher, USA), PE-Cyanine7-labeled anti-CD4 antibody (eBioscience, USA) and PerCP-Cyanine5.5-labeled anti-CD8α antibody (eBioscience, USA) were added. The proliferation of immune cells was evaluated by flow cytometry.

### Enzyme-linked immunospot assays (ELISPOTs) for TNF-α and IFN-γ

TNF-α and IFN-γ concentrations were measured with mouse TNF-α and IFN-γ ELISPOT kits (Mabtech, Sweden), respectively, according to the vendor’s instructions. A total of 2.5 × 10^5^ activated NK cells or T cells per well were plated in an anti-cytokine mAb-coated plate overnight. The spots per well were visualized and recorded automatically by a spot counter (EliSpot Reader-iSpot, Germany). Cytokines secreted by NK cells or T cells were evaluated by counting the of spots in wells.

### Cytotoxicity assay

The cytotoxicity of effector NK cells or T cells to target cells was tested with a CytoTox 96® Non-Radioactive Cytotoxicity Assay (Promega, Madison, WI, USA). BP210 or BP210-T315I cells were chosen as target cells. First, effector NK cells or T cells prestimulated with PBS or exosomes were mixed with 1 × 10^4^ target cells in each well at different designated ratios. Then, half of the supernatant of each sample was extracted and incubated with a substrate solution. The amount of released lactate dehydrogenase (LDH) was calculated and considered to reflect the cytotoxicity of the immune cells.

### Enzyme-linked immunosorbent assay (ELISA) for IL-2

The culture supernatants of T lymphocytes prestimulated with exosomes for 36 h were harvested to detect the concentration of IL-2. The IL-2 level of T lymphocytes from each group was examined with mouse IL-2 ELISA kits (Mabtech, Sweden) following the manufacturer’s recommendations.

### Generation of murine models

Six to eight-week-old Balb/c mice were housed in a specific pathogen-free environment in the animal unit of Chongqing Medical University. Mouse CML models were established by intravenous injection of 3 × 10^6^ BP210 or BP210-T315I cells suspended in PBS. Seven days after CML cell inoculation, the mice were injected intradermally with 50 μg of exosomes per mouse or an equivalent volume of PBS every second day for four weeks. Body weight and the peripheral white blood cell (WBC) count were measured weekly. Weight loss, ruffled fur, hunched posture, abnormal leukocytosis, and classic indicators of extramedullary hematopoiesis such as hepatosplenomegaly were considered as the typical CML-like disease characteristics and recorded during an observation period of 90 days.

To test whether B-R-Dex or BT-R-Dex could exert durable antitumor effects in vivo, mice in the groups that survived the treatment cycle received a second injection of 3 × 10^6^ BP210 or BP210-T315I cells via the tail vein. New naive Balb/c mice that were age- and sex-matched were used as control groups and intravenously injected with the same number of BP210 or BP210-T315I cells. The typical CML-like disease characteristics were also recorded during a 90-day observation period.

All the in vivo experiments described were conducted with the approval of the Institutional Animal Care and Ethical Committee of Chongqing Medical University.

### Immunofluorescence assay

Tissue smears were incubated with 4% paraformaldehyde followed by permeabilization with prewarmed 0.1% Triton X-100. Cells were blocked with ready-to-use goat serum (BOSTER, China) and then incubated with a primary antibody (Santa Cruz Biotechnology, USA) overnight. Afterward, the cells were incubated with a corresponding fluorescently labeled anti-mouse secondary antibody (Thermo Fisher Scientific, USA) for 60 min. Finally, nuclei were stained with DAPI as described above. Slides were examined under a fluorescence microscope.

### Statistical analysis

Data are reported as the mean ± standard deviation (SD). GraphPad Prism 8 software and SPSS (Version 19.0) software were used for all statistical tests and graph generation. The statistical significance of differences among multiple groups was analyzed by one-way ANOVA. Student’s t test was used to evaluate differences between two groups. The Kaplan–Meier method and log-rank (Mantel–Cox) test were employed to compare survival differences. Two-tailed P values < 0.05 were considered significant.

## Results

### BP210-RAE-1γ-Dex and BP210-T315I-RAE-1γ-Dex are successfully prepared by loading DCs with lysates of CML cells expressing RAE-1γ

**Fig. 1:**
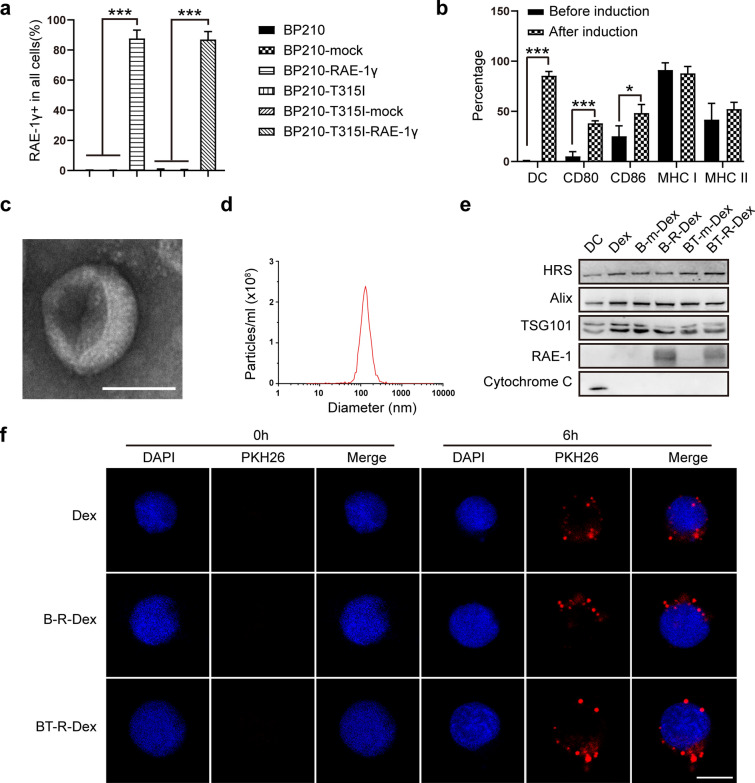
CML-RAE-1γ-Dex were isolated from DCs loaded with lysates of RAE-1γ-expressing CML cells. **a** Flow cytometric analysis of the expression of RAE-1γ in parental and transfected CML cells. **b** Flow cytometric analysis of the percentages of cells with positive expression of CD11c, CD80, CD86 and MHC class I/II before and after cytokine induction. **c** The morphology of Dex was visualized by TEM. Scale bar, 100 nm. **d** The particle size of Dex was measured by NTA. **e** The expression of HRS, Alix, TSG101, Cytochrome C, and RAE-1 in DCs and various types of Dex was measured by western blot analysis. **f** NK cells were incubated with Dex labeled with PKH26 (red). After fixation, the nuclei were stained with DAPI (blue). Finally, representative images were obtained by confocal microscopy. Red fluorescent particles represent PKH26-labeled Dex. Scale bar, 5 μm. Values are presented as the mean ± SD. *p < 0.05, ***p < 0.001

Among the five RAE-1 isoforms (RAE-1α, RAE-1β, RAE-1γ, RAE-1δ and RAE-1ε), RAE-1γ, which has high affinity for NKG2D, can induce robust NK-cell and T-cell-mediated immune responses [34, 35]. To prepare RAE-1γ-expressing tumor cell lysates from CML cells, BP210 cells and imatinib-resistant BP210-T315I cells were transfected with lentiviruses encoding RAE-1γ. The lentivirus-mediated expression of RAE-1γ in BP210-RAE-1γ and BP210-T315I-RAE-1γ cells was detected by flow cytometry. Compared with matched controls, both BP210-RAE-1γ cells and BP210-T315I-RAE-1γ cells expressed a high level of RAE-1γ (Fig. 1a and Additional file 1: Fig. S1a). Then we generated tumor cell lysates from BP210-RAE-1γ and BP210-T315I-RAE-1γ cells, which were used as tumor antigens for DC loading.

Isolated Balb/c BMDCs were induced with cytokines and then evaluated by flow cytometry. First, the phenotypic characteristics and proportions of DC subsets were identified according to the surface biomarkers CD11c, CD80, CD86, and MHC class I/II. High expression of costimulatory molecules such as CD80 and CD86 indicates a mature DC phenotype and contributes to immune activation [39, 40]. MHC class I and II are also involved in efficient antigen presentation [41]. Cytokine treatment increased the percentage of DCs and the levels of CD80 and CD86 compared with no treatment (Fig. 1b and Additional file 1: Fig. S1b). These results showed that DCs were successfully induced and supported the collection of Dex for further experiments.

We purified Dex from the culture medium of DCs after loading with whole CML antigens and inducing maturation. To verify the characteristics of Dex, exosomal morphology and the size distribution were detected by TEM and NTA, respectively. Typical cup-shaped particles were visualized by TEM (Fig. 1c), and the diameter detected by NTA ranged from 30 to 150 nm (Fig. 1d), which was consistent with the biological characteristics of exosomes [20]. Representative exosomal markers, including HRS, Alix, and TSG101, were detected by immunoblotting. Additionally, cytochrome C, a mitochondria protein, was used as a negative maker for exosomes. As shown in Fig. 1e, the Dex composition did not change after loading with various types of whole cell lysates. Furthermore, BP210-RAE-1γ-Dex (B-R-Dex) and BP210-T315I-RAE-1γ-Dex (BT-R-Dex), which were termed CML-RAE-1γ-Dex, contained a high level of RAE-1. To measure the uptake of Dex by NK cells, NK cells were coincubated with PKH26-labeled Dex for 6 h. Immunofluorescence imaging demonstrated that Dex accumulated in NK cells, indicating that the generated Dex could bind to NK cells (Fig. 1f).

### NK cells targeting BP210 cells or BP210-T315I cells can be activated by BP210-RAE-1γ-Dex or BP210-T315I-RAE-1γ-Dex in vitro

**Fig. 2:**
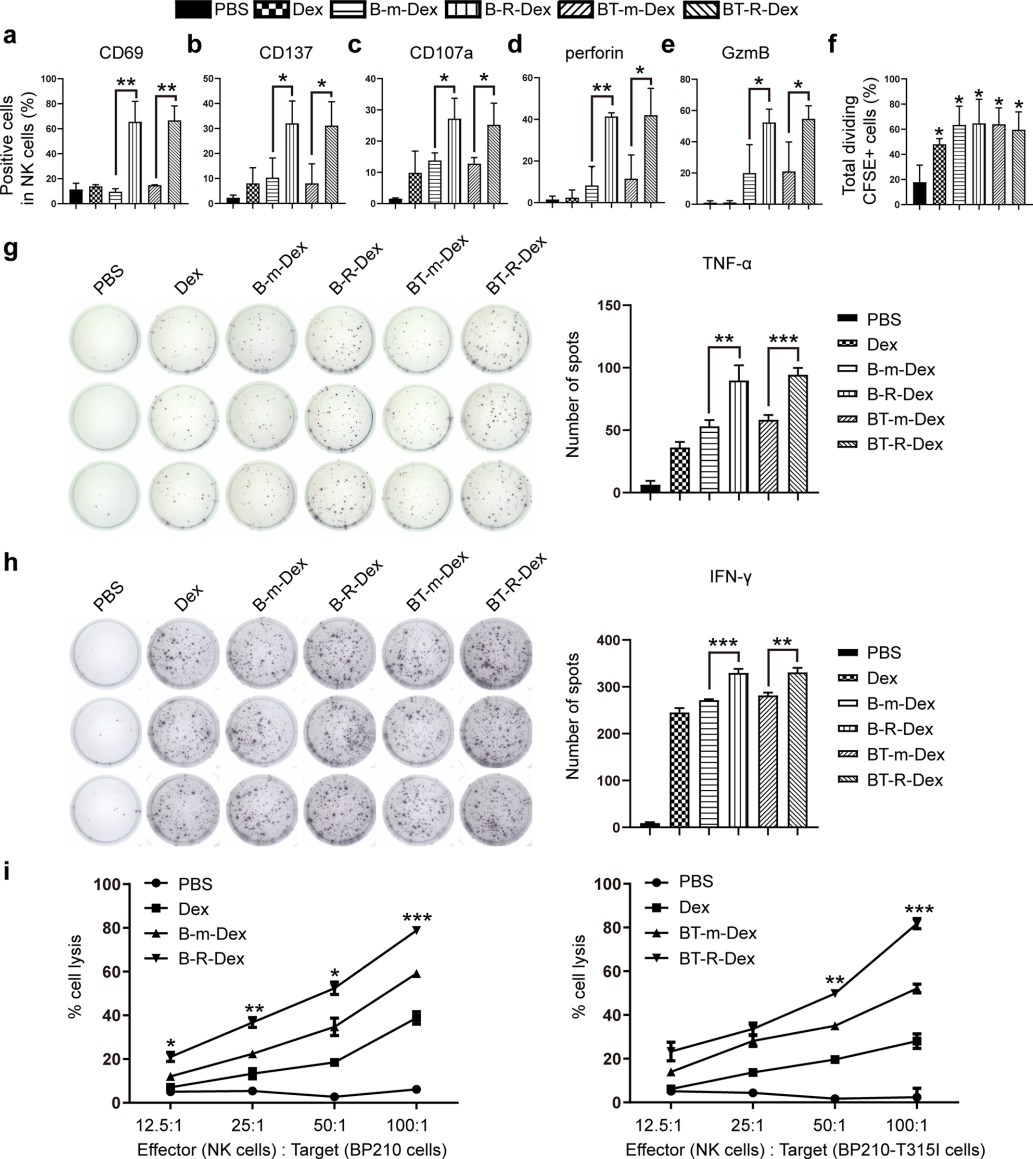
CML-RAE-1γ-Dex promoted NK-cell activation and proliferation. a-e Flow cytometric analysis of CD69 (**a**), CD137 (**b**), CD107a (**c**), perforin (**d**) and GzmB (**e**) expression in NK cells after exposure to exosomes for 6 h. **f** NK-cell proliferation after 72 h of culture with Dex was evaluated by flow cytometric analysis. *p < 0.05 vs. the PBS group. **g**, **h** NK cells were treated with exosomes for 6 h and then seeded in ELISPOT plates overnight. ELISPOT assays were employed to compare TNF-α and IFN-γ production by activated NK cells among the groups. **i** A cytotoxicity assay was used to measure the killing ability of effector NK cells prestimulated with exosomes for 6 h against BP210 (left) or BP210-T315I (right) target cells. Cytotoxic activity was detected at the effector-to-target (E:T) ratios of 12.5:1, 25:1, 50:1, and 100:1, respectively. Values are presented as the mean ± SD. *p < 0.05, **p < 0.01, ***p < 0.001 vs. the mock group

NK-cell activation and expansion are critical determinants of immunotherapeutic effects [42]. Thus, we detected the effects of CML-RAE-1γ-Dex on the activation and proliferation of NK cells. It is known that the expression of CD69 and CD137 (4-1BB), which are members of the tumor necrosis factor (TNF)-receptor superfamily, can be induced on activated T lymphocytes and NK cells and that these molecules are classic activation biomarkers [43, 44]. First, we measured the levels of CD69 and CD137 on NK cells by flow cytometry. As shown in Fig. 2a, b and Additional file 2: Fig. S2a–b, NK cells cocultured with Dex, BP210-mock-Dex (B-m-Dex) or BP210-T315I-mock-Dex (BT-m-Dex) expressed low levels of CD69 and CD137. In contrast, B-R-Dex increased the expression of CD69 and CD137 on NK cells. Similar results were also observed for NK cells treated with BT-R-Dex. These results demonstrated that NK cells were strongly activated by CML-RAE-1γ-Dex. Second, to further investigate the cytolytic activity of NK cells, we evaluated the production of the classic degranulation marker CD107a (lysosome-associated membrane protein-1, LAMP-1), as well as that of perforin and granzyme B (GzmB) which are potential cytolytic biomarkers of NK cells and T lymphocytes [45–47] by flow cytometry after coculturing NK cells with CML-RAE-1γ-Dex or various controls. Compared to PBS, Dex and B-m-Dex, B-R-Dex enhanced degranulation, and the generation of perforin and GzmB (Fig. 2c–e and Additional file 2: Fig. S2c–e) in NK cells. A similar pattern was also observed for NK cells stimulated with BT-R-Dex. These flow cytometry results suggested that B-R-Dex and BT-R-Dex treatments displayed a greater capacity to promote the cytotoxic activity of NK cells. Finally, a CFSE-based assay was adopted to assess the proliferative activity of immune cells. More extensive proliferation was observed at 72 h for NK cells exposed to naked Dex, CML-mock-Dex or CML-RAE-1γ-Dex than for NK cells in the PBS control group (Fig. 2f and Additional file 2: Fig. S2f). Our experimental data were consistent with previous research showing Dex could induce NK-cell proliferation [27]. Notably, no significant differences in the capacity to promote leucocyte expansion and activation were observed between B-R-Dex and BT-R-Dex. All NK-cell populations (CD3^−^DX5^+^) were gated for flow cytometric analysis (Additional file 2: Fig. S2g). In conclusion, CML-RAE-1γ-Dex effectively enhanced the effector functions and proliferation of NK cells.

In addition to flow cytometric detection, further study was performed to evaluate NK-cell function, including investigation of the release of effector cytokines such as TNF-α and IFN-γ [42] after exposure to modified Dex by ELISPOT assays. First, the purity of Balb/c NK cells (CD3^−^ DX5^+^ cells) enriched by DX5-MicroBead selection was approximately 85.5% (Additional file 2: Fig. S2h). We cocultured these NK cells with exosomes and then measured TNF-α and IFN-γ production. In this experiment, B-R-Dex markedly increased the release of TNF-α (Fig. 2g) and IFN-γ (Fig. 2h) compared to PBS, Dex and B-m-Dex. Similarly, the NK cells in the BT-R-Dex group also exhibited greater secretion of effector cytokines. We noticed that naked Dex did not exert an obvious effect on the release of perforin and GzmB by NK cells (Fig. 2d, e) but induced moderate increases in TNF-α and IFN-γ (Fig. 2g, h). The possible explanations were that the different detection time of the experiments or the purity of isolated NK cells caused this discrepancy in the results. Additionally, the intracellular flow cytometry assay is less sensitive than the ELISPOT assay, especially for weaker immune response [48, 49]. Then, NK-cell cytotoxicity induced by different stimuli was compared with an LDH assay. The strong dependence of NK-cell reactivity on NKG2D-RAE-1γ binding in this setting was revealed by the higher lysis rate for CML-RAE-1γ-Dex groups than for CML-mock-Dex groups (Fig. 2i). Notably, NK cells treated with BT-R-Dex exerted a killing effect on BP210-T315I cells, a CML cell line carrying the T315I resistance mutation. The LDH analysis indicated that NK cells treated with modified Dex were effective and functional against drug-resistant CML cell lines. Consequently, these findings confirmed that stimulation with CML-RAE-1γ-Dex could help NK cells achieve strong anti-CML efficacy.

### T-cell cytotoxicity to BP210 cells and BP210-T315I cells is induced by BP210-RAE-1γ-Dex or BP210-T315I-RAE-1γ-Dex in vitro

**Fig. 3:**
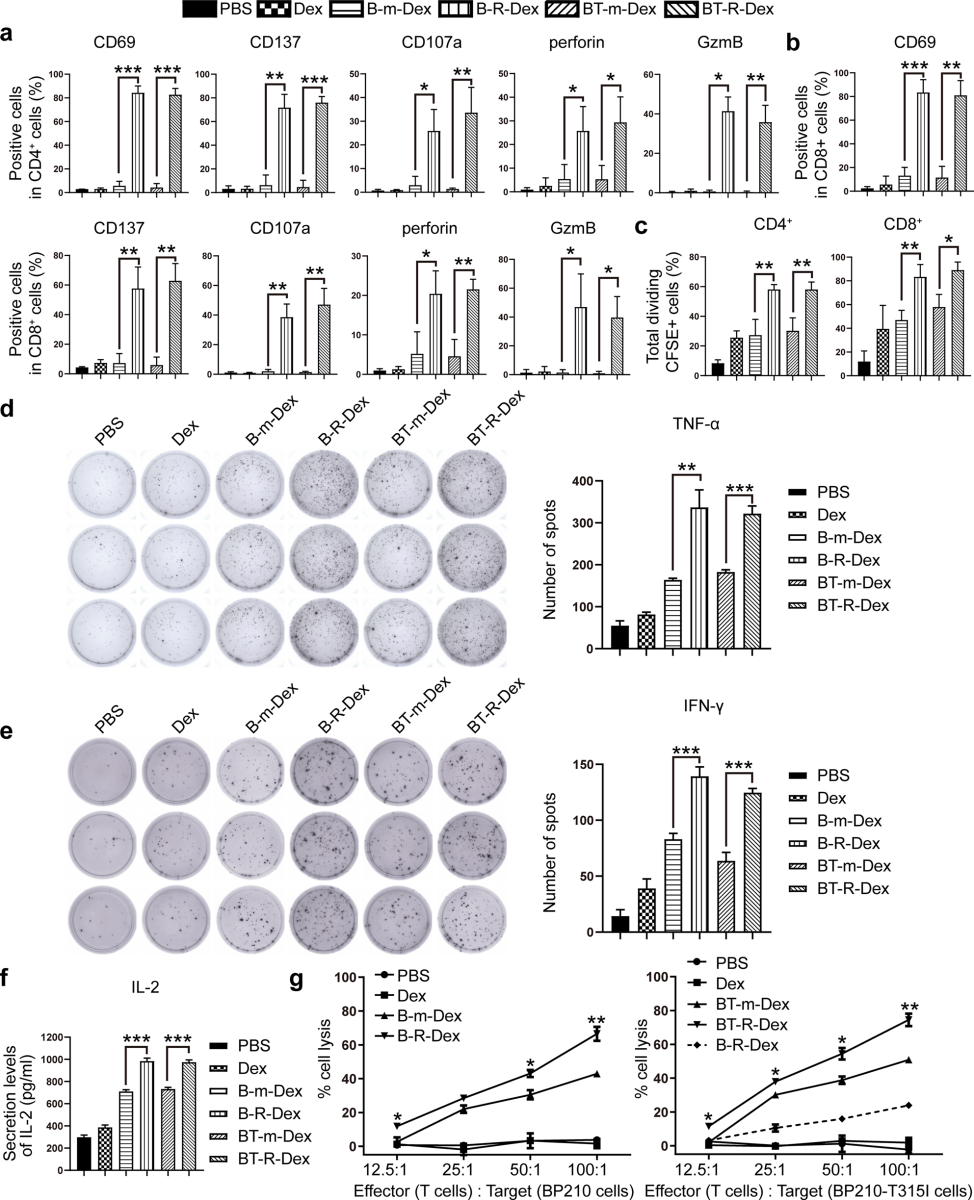
Immunostimulatory effect of Dex on T lymphocytes in vitro. **a** The proportions of CD69^+^, CD137^+^, CD107a^+^, perforin^+^ and GzmB^+^ cells in CD4^+^ T subpopulations after 36 h of incubation with PBS or exosomes were assessed by flow cytometry. **b** Effects of Dex on activation biomarkers, degranulation activity, and cytotoxic mediators including perforin and GzmB in CD8^+^ T lymphocytes after 36 h of incubation. **c** The proliferative ability of CD4^+^ and CD8^+^ T lymphocytes was assessed by CFSE-based analysis after coculture with PBS or exosomes for 72 h. **d**, **e** T lymphocytes were pretreated with exosomes. After 36 h, the T cells were seeded in ELISPOT plates overnight. The secretion of TNF-α (**d**) or IFN-γ (**e**) secreted by activated T lymphocytes was assessed by ELISPOT assays. **f** T cells were cocultured with PBS or exosomes for 36 h. ELISA was carried out to measure the concentration of IL-2 (pg/ml) in the supernatant of the T cells. **g** The cytotoxic effects of T lymphocytes pretreated with exosomes for 36 h were tested by an LDH assay at different E:T ratios (12.5:1, 25:1, 50:1, and 100:1). BP210 or BP210-T315I cells were used as the target cells. Values are presented as the mean ± SD. *p < 0.05, **p < 0.01, ***p < 0.001 vs. the mock group

RAE-1 can bind to the murine immune-activating receptor NKG2D and transduce a costimulatory signal that promotes the TCR-dependent activation of T cells [33, 50], which suggests that engagement of NKG2D/RAE-1γ may augment both the cytokine production and cellular proliferation of murine T cells. To test whether CML-RAE-1γ-Dex can modulate T-cell activity and killing efficiency, we cocultured freshly isolated murine T cells with different types of Dex and evaluated their activation and killing capacity. Representative activation biomarkers including CD69 and CD137 [43, 44], and cytotoxicity indicators such as CD107a, perforin and GzmB [45–47] of T lymphocytes were measured by flow cytometry. Compared with B-m-Dex treatment, B-R-Dex treatment significantly upregulated CD69, CD137, CD107a, perforin and GzmB expression in CD4^+^ and CD8^+^ T cells (Fig. 3a, b and Additional file 3: Figs. S3a-e and S4a-e). Similar to B-R-Dex, BT-R-Dex also induced high percentages of cells expressing CD69, CD137, CD107a, perforin and GzmB in CD4^+^ and CD8^+^ T cells. Consistent with the previous results for NK cells, CD4^+^ T cells and CD8^+^ T cells could be activated CML-RAE-1γ-Dex treatment. Then, a CFSE labeling assay was performed to detect the proliferative capacity of T lymphocytes. As the flow cytometry data indicated, both B-R-Dex and BT-R-Dex induced the strong proliferation of CD4^+^ T cells and CD8^+^ T cells relative to the corresponding controls (Fig. 3c, and Additional file 3: Fig. S3f, Additional file 4: Fig. S4f). Total CD4^+^ and CD8^+^ T lymphocytes were determined based on the immunophenotypic markers CD3, CD4, and CD8 (Additional file 3: Fig. S3g). The results indicated that an immunity response mediated by T lymphocytes was induced and enhanced, showing increasing numbers of T-cell populations, following stimulation with B-R-Dex or BT-R-Dex.

To further support the functional activation of T lymphocytes by CML-RAE-1γ-Dex, in addition to flow cytometric detection, ELISPOT assays were used to assess the secretion of cytotoxic cytokines including TNF-α and IFN-γ [51], and ELISA was performed to assess the production of IL-2, a critical cytokine that is synthesized by activated T cells [51]. As shown in Fig. 3d, e, T lymphocytes produced large amounts of TNF-α and IFN-γ following B-R-Dex stimulation. A similar trend was also shown for the BT-R-Dex group. Furthermore, in comparison with the respective controls, namely, T lymphocytes in the B-m-Dex and BT-m-Dex groups, T lymphocytes in the B-R-Dex and BT-R-Dex groups secreted significantly higher levels of IL-2 (Fig. 3f). These data further confirmed that B-R-Dex and BT-R-Dex notably elicited immune response by lymphocytes. Flow cytometric analysis revealed that effective T-cell immune responses were not induced by Dex or CML-mock-Dex (Fig. 3a, b), in contrast to enhancement of T-cell effector functions induced by CML-mock-Dex shown by the ELISA and ELISPOT assays (Fig. 3d–f). The reasons for this discrepancy could depend on the different detection time among the distinct experiments, the low purity of T lymphocytes or the different detection sensitivities of these methods [48]. We next compared the cytotoxicity of T lymphocytes across different groups. The CML cell lines BP210 and BP210-T315I (target cells) were mixed with activated T lymphocytes (effector cells) at multiple effector-target cell (E:T) ratios. Significant increases in LDH release were observed in the B-R-Dex and BT-R-Dex groups compared with the corresponding control groups (Fig. 3g). Notably, at an E/T ratio of 100 to 1, T lymphocytes activated by CML-RAE-1γ-Dex exhibited significant killing activity against CML cells, which was increased to nearly 80%, as compared with those primed by antigen-loaded Dex alone. The high cytolytic activity of T cells stimulated with BT-R-Dex against BP210-T315I cells suggested a potential avenue to solve problems related to drug-resistance. Additionally, despite increasing the E:T ratio, T cells stimulated with B-R-Dex still had a lower cytotoxic effect on BP210-T315I cells, indicating that Dex pulsed with distinct antigens from whole tumor cells were able to prime antigen-specific immune responses. Furthermore, compared to CML-RAE-1γ-Dex, CML-mock-Dex induced only limited T-cell responses. This result suggested that CML-RAE-1γ-Dex overcame the limited antigen presentation capacity and poor immunogenicity of antigen-loaded Dex alone. Overall, CML-RAE-1γ-Dex showed predominant immunostimulatory effects, implying that RAE-1γ enhanced Dex-based immunogenicity in the presence of whole tumor cell lysates.

### CML-RAE-1γ-Dex-mediated immunostimulatory activity depends on the NKG2D/NKG2D-L pathway

**Fig. 4:**
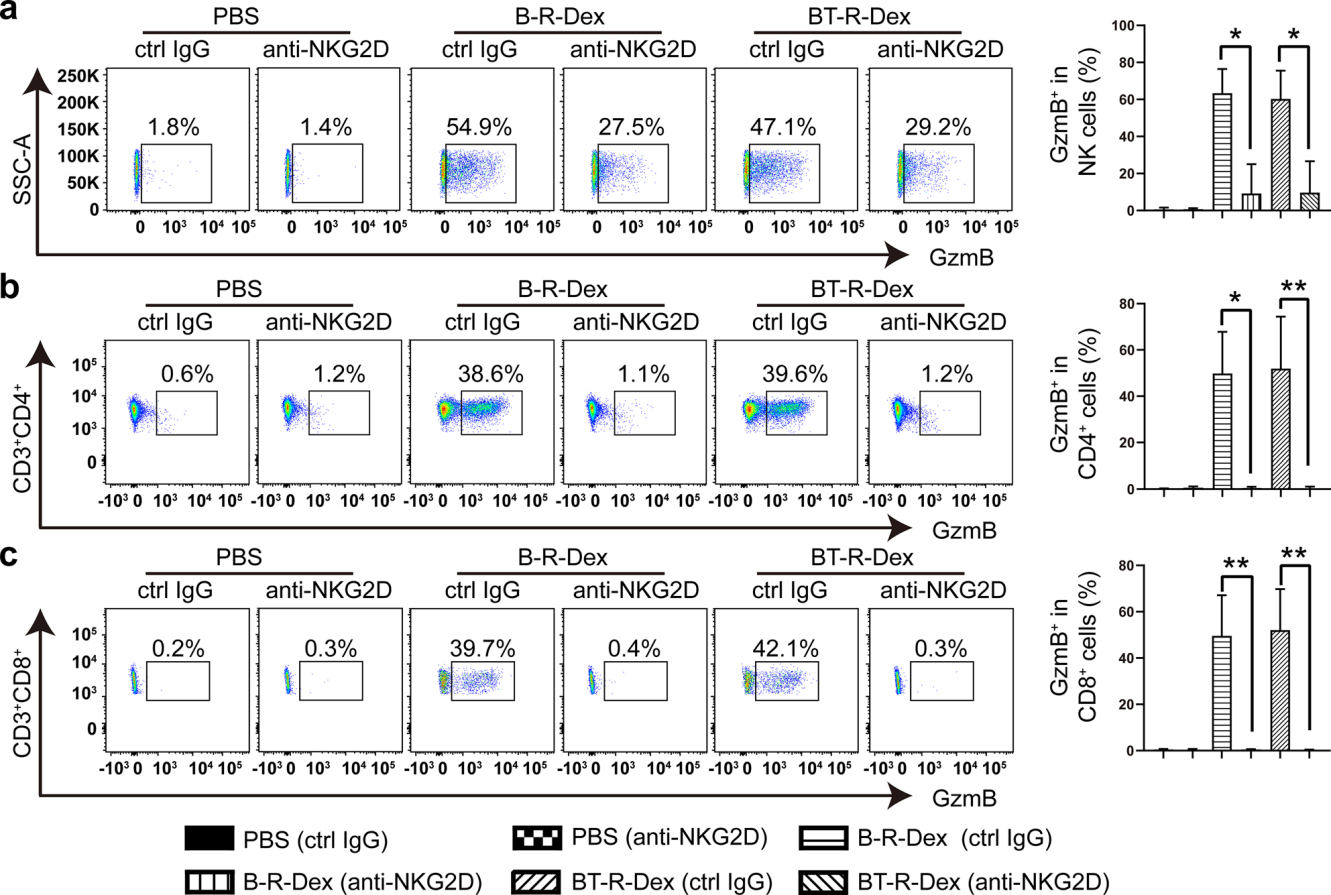
NKG2D-dependent activation and GzmB production of NK cells and T lymphocytes. NK cells or T lymphocytes were pretreated with an anti-NKG2D antibody or a control IgG antibody for 30 min and then stimulated with exosomes for 6 h and 36 h, respectively. **a–c** The intracellular expression of GzmB in NK cells (**a**), CD4^+^ T cells (**b**) and CD8^+^ T cells (**c**) was measured by intracellular staining and flow cytometric analysis. Representative flow cytometry plots showing the expression level of intracellular GzmB in these immune cells. Values are presented as the means ± SD. *p < 0.05, **p < 0.01 vs. the corresponding ctrl IgG group

NKG2D specifically binds to NKG2D-L to transmit an activation signal and elicit effector immune cells that can eliminate tumor cells [52]. Moreover, based on our observations that CML-RAE-1γ-Dex possessed higher immunostimulatory activity than CML-specific Dex without RAE-1γ modification, we next wanted to investigate whether the NKG2D/NKG2D-L (RAE-1γ) pathway is a crucial determinant in NK/T-cell activation induced by modified Dex. A neutralizing anti-mouse mAb against NKG2D was employed to block the interaction between NKG2D and NKG2D-L [53]. NK cells and T lymphocytes isolated from Balb/c mouse spleens were first preincubated with the anti-NKG2D mAb or a control IgG (ctrl IgG) before coculture with CML-RAE-1γ-Dex or PBS. The cytotoxic potential of immune cells was detected by flow cytometric analysis of the proportion of GzmB-positive cells. As depicted in Fig. 4a, NKG2D blockade significantly reduced GzmB production by NK cells in the B-R-Dex group or BT-R-Dex group. Consistent with these data, blocking NKG2D in T cells impaired NKG2D-mediated cytotoxicity by decreasing GzmB production (Fig. 4b, c), and ultimately inhibited CML-RAE-1γ-Dex-induced T-cell responses. In summary, CML-RAE-1γ-Dex contributed to immune function in a NKG2D/NKG2D-L (RAE-1γ)-dependent manner, as blocking NKG2D signaling with a neutralizing antibody against NKG2D reduced the cytotoxicity of NK cells and T lymphocytes.

### CML-RAE-1γ-Dex suppress the development of CML induced by BCR-ABL or BCR-ABL^T315I^ in vivo

**Fig. 5:**
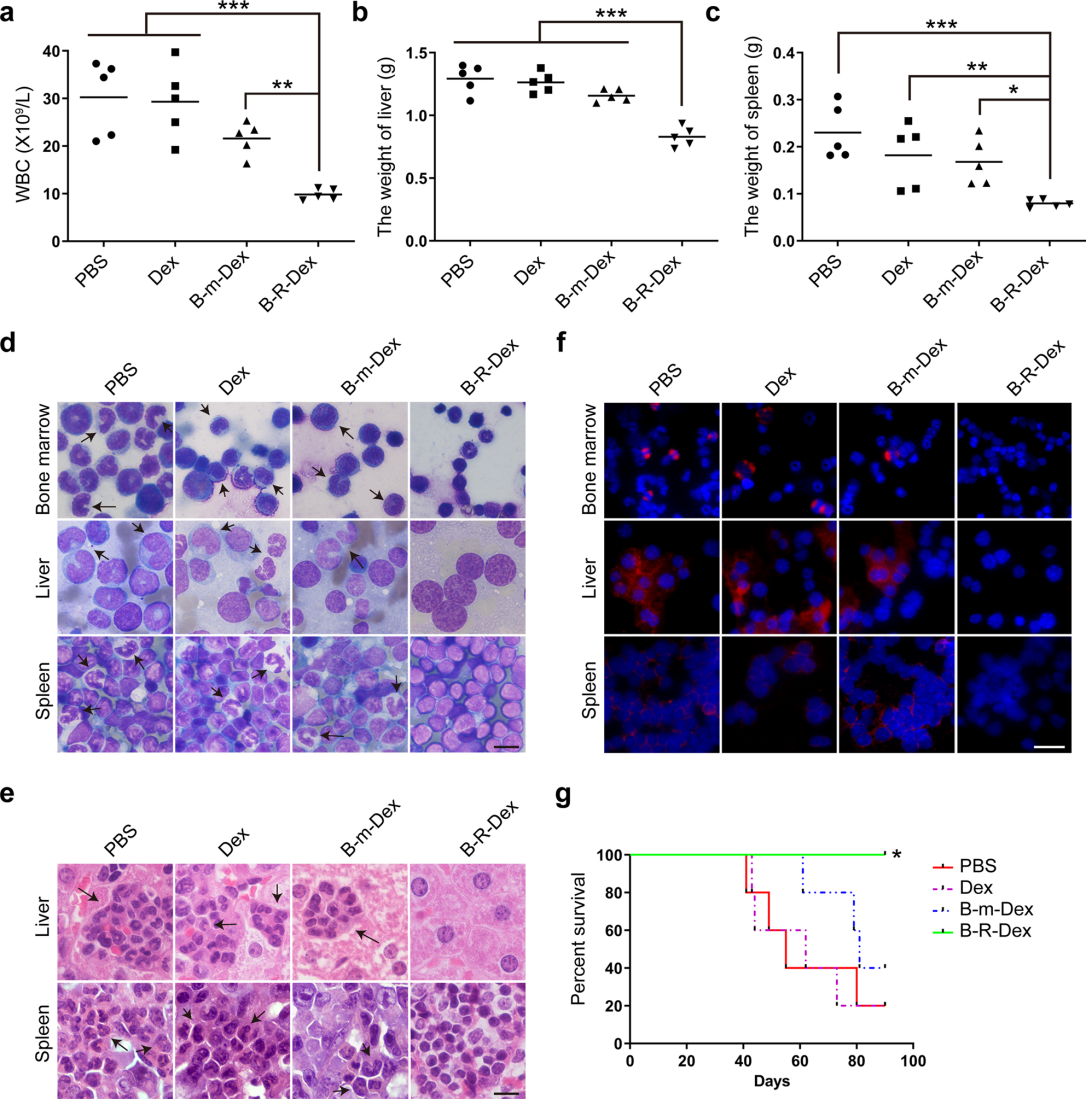
Therapeutic effects of BP210-RAE-1γ-Dex in a murine BP210 tumor model. **a** The average of the maximum WBC count of every group was calculated. **b**, **c** The liver (**b**) and spleen (**c**) were harvested and weighed. **d** Representative images of tissue smears stained with Wright’s staining are shown. The black arrows point to abnormal cells invading tissues. Scale bar, 10 μm. **e** Hematoxylin and eosin-stained liver and spleen tissues were used to confirm pathological characteristics. The arrows indicate leukemic cells. Scale bar, 10 μm. **f** Immunofluorescence assays were conducted to determine the level of BCR-ABL expression. Scale bar, 25 μm. **g** Kaplan–Meier survival analysis was performed to analyze the survival times of treated mice. *p < 0.05, **p < 0.01, ***p < 0.001

**Fig. 6:**
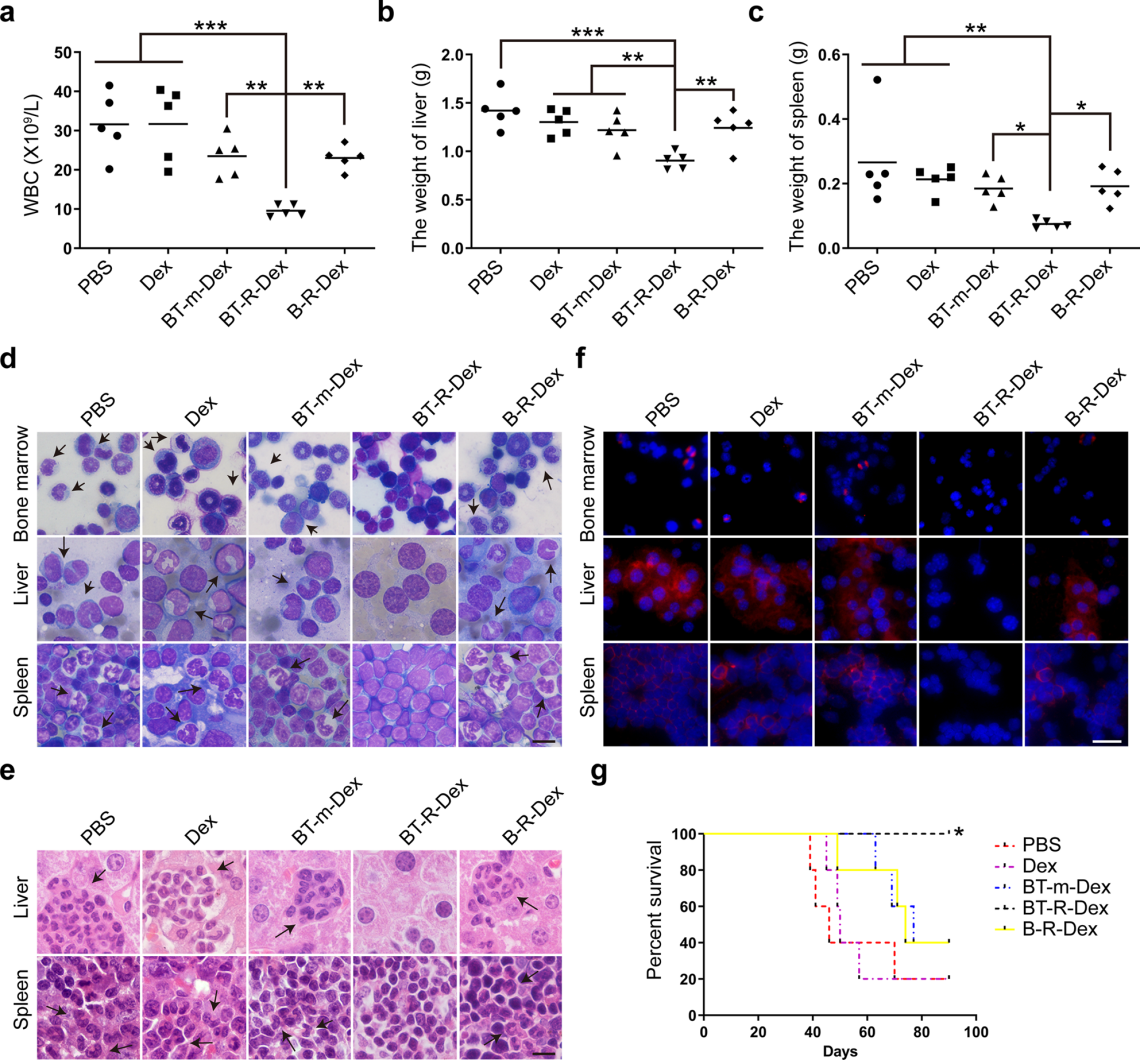
Anti-tumor immune responses induced by BP210-T315I-RAE-1γ-Dex in the BCR-ABL^T315I^-induced CML mouse model. **a** Peripheral WBC counts were analyzed. **b**, **c** The weights of the liver (**b**) and spleen (**c**) were recorded. **d** Wright’s staining showed abnormal leukemic cells (arrows) in bone marrow, spleen and liver tissues. Scale bar, 10 μm. **e** Histological sections stained with H&E showed the infiltration of leukemic cells (arrows). Scale bar, 10 μm. **f** The BCR-ABL oncoprotein was identified with a fluorophore-labeled antibody in the liver and spleen and observed under a microscope. Scale bar, 25 μm. **g** The survival rates of tumor-bearing mice were measured by Kaplan–Meier methods. *p < 0.05, **p < 0.01, ***p < 0.001

To investigate whether modified Dex can exert therapeutic effects in vivo, murine leukemogenesis models were established by injection of 3 × 10^6^ CML cells into Balb/c mice via the tail vein. Some mice were inoculated with BP210 cells and used as a CML model induced by BCR-ABL, while others were inoculated with BP210-T315I cells and used as an imatinib-resistant CML model induced with BCR-ABL^T315I^. One week after tumor cell inoculation, the mice received intradermal injections of naked Dex, CML-mock-Dex, CML-RAE-1γ-Dex or an equivalent volume of PBS. The WBC count in the peripheral blood was monitored weekly, and the maximum WBC count was recorded. As shown in Fig. 5a–c and Fig. 6a–c, B-R-Dex or BT-R-Dex markedly decreased the levels of leukocytes and the weights of the liver and spleen in mice while the opposite results were found for mice in the PBS, Dex, B-m-Dex and BT-m-Dex treatment groups. Consistent results were also observed by comparing splenomegaly and hepatomegaly among the mice in the different treatment group (Additional file 5: Fig. S5a, b). Wright’s staining results showed that the bone marrow, liver and spleen tissues of mice treated with PBS, Dex, or CML-mock-Dex were severely infiltrated with leukemic cells, whereas reduced amounts of leukemic cells were observed in these organs in mice treated with B-R-Dex or BT-R-Dex (Figs. 5d and 6d). Similarly, hematoxylin and eosin (H&E) staining results verified that compared to the control treatments, both B-R-Dex administration and BT-R-Dex administration strongly inhibited leukemic cell infiltration in the liver and spleen (Figs. 5e and 6e). Additionally, the expression level of BCR-ABL was investigated in the bone marrow, liver and spleen by immunofluorescence staining. B-R-Dex or BT-R-Dex treatment markedly suppressed the expression of the oncoprotein BCR-ABL in these tissues compared with PBS, Dex, or CML-mock-Dex treatment (Figs. 5f and 6f). All these results indicated that treatment with B-R-Dex or BT-R-Dex efficiently suppressed leukemia cell propagation and infiltration. Kaplan–Meier survival analysis indicated that tumor-bearing mice receiving B-R-Dex or BT-R-Dex administration showed significantly extended overall survival compared to those in the other groups (Figs. 5g and 6g). Moreover, we noticed that B-R-Dex could not prevent the malignant progression of CML in the BP210-T315I mouse model. In this model, B-R-Dex treated mice showed an increased WBC, hepatosplenomegaly, severe leukemic infiltration and a shorter survival period. In contrast, BT-R-Dex produced a satisfactory therapeutic effect in this mouse model, suggesting that CML-RAE-1γ-Dex may elicit an antigen-specific antitumor effect (Fig. 6). In conclusion, CML-RAE-1γ-Dex treatment inhibited the occurrence and development of CML induced by BCR-ABL with or without the T315I mutation, leading to a notable improvement in the overall survival of CML mice.

### CML-RAE-1γ-Dex exert a long-term therapeutic effect on BCR-ABL and BCR-ABL^T315I^ -induced CML in vivo

**Fig. 7:**
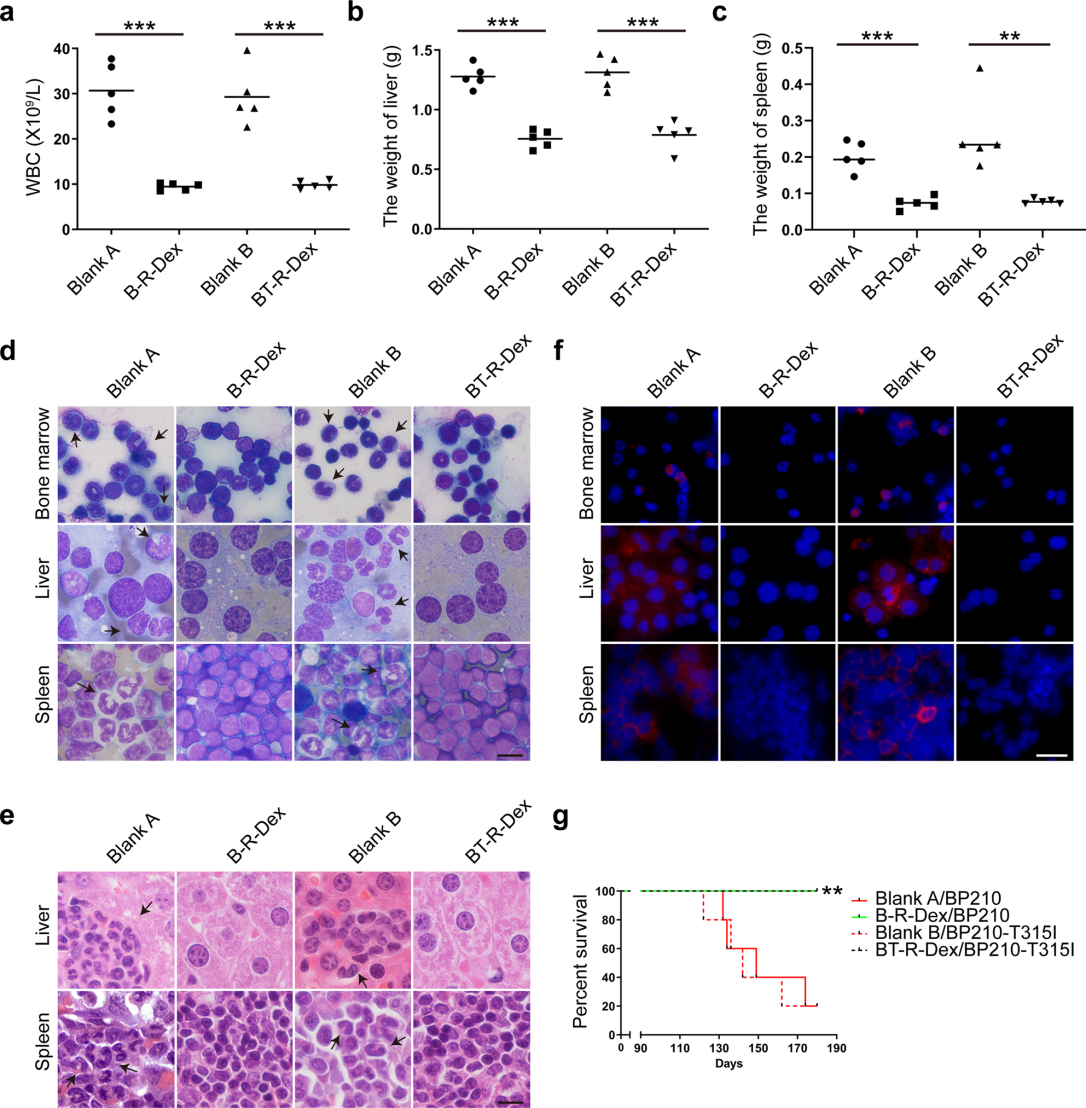
Long-term immunological effect of CML-RAE-1γ-Dex in vivo. Blank A and Blank B were inoculated with BP210 and BP210-T315I cells, respectively. **a** The maximum WBC count was recorded. **b**, **c** The weights of the liver (**b**) and spleen (**c**) in the four groups were measured. **d** Wright’s staining was carried out to examine leukemic cell infiltration in tissues. Microscopy images show the typical morphology of leukemic cells (arrows). Scale bar, 10 μm. **e** Tissue sections from the liver and spleen were stained H&E and showed infiltrated leukemic cells. Scale bar, 10 μm. **f** Cross-sectional microscopy images showing immunofluorescence staining were used to evaluate the BCR-ABL protein in bone marrow, spleen and liver tissues. Scale bar, 25 μm. **g** The Kaplan–Meier survival method was conducted to compare differences in survival among groups. **p < 0.01, ***p < 0.001

Encouraged by the robust antitumor immune responses induced by CML-RAE-1γ-Dex, we explored whether these modified Dex could elicit a durable immune response in vivo. To investigate long-term immune efficacy of CML-RAE-1γ-Dex against myeloid leukemia, we rechallenged surviving mice in the B-R-Dex and BT-R-Dex treatment groups with the same number of BP210 or BP210-T315I cells, respectively. Naive mice that were age- and sex-matched were inoculated with tumor cells and used as controls. As shown in Fig. 7a–c and Additional file 5: Fig. S5c, abnormal leukocytosis and uncontrollable hepatomegaly and splenomegaly were observed in the control groups, but not in the B-R-Dex and BT-R-Dex groups. These results demonstrated that the survivors treated with B-R-Dex or BT-R-Dex vaccination exhibited notable immune protection against secondary exposure to CML cells. Furthermore, as shown in Fig. 7d, e, Wright’s and H&E staining analyses confirmed reduced leukemic infiltration in the bone marrow, liver and spleen in the immunized groups. Additionally, consistent results were obtained by comparing the expression levels of the BCR-ABL oncoprotein in various tissues among the groups (Fig. 7f). As expected, treatment with B-R-Dex or BT-R-Dex markedly improved the survival time of mice rechallenged with leukemia cells compared with no vaccination (Fig. 7g). Altogether, these results further revealed that CML-RAE-1γ-Dex vaccines induced a robust anamnestic response and provided long-term protection against leukemia.

## Discussion

**Fig. 8:**
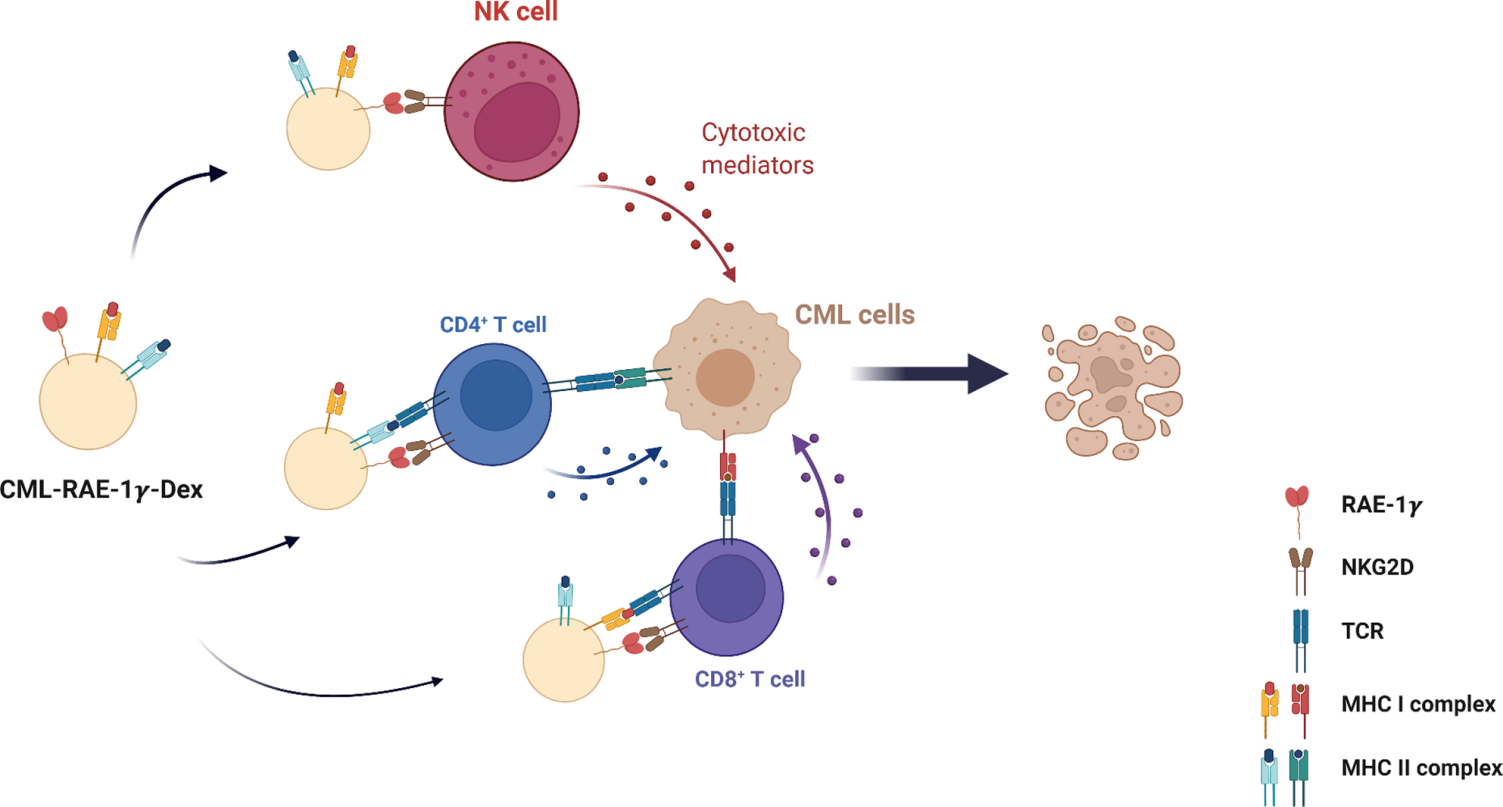
Schematic illustration of CML-RAE-1γ-Dex-based cancer immunotherapy. CML-RAE-1γ-Dex augment NK-cell and CD4^+^ and CD8^+^ T-cell activation in an NKG2D-dependent manner and amplify the immune response by promoting NK/T-cell proliferation. Created with BioRender.com

Chronic myeloid leukemia is a malignant hematological tumor with a characteristic chimeric fusion protein, with an incidence of approximately 2/100,000, and poses a serious threat to patients’ lives [54]. The first-generation TKI imatinib is regarded as the first choice for patients with CML. However, imatinib resistance involving the T315I mutation in the ABL-kinase domain is a main challenge in CML therapy [4]. Although third-generation TKIs have been developed and subsequently approved by the Food and Drug Administration (FDA) and have shown potent against the T315I mutation, their application in the clinic is restricted due to unacceptable side effects [5]. Disease progression and imatinib resistance are still the major obstacles in the treatment of CML patients. Hence, exploration of more effective and feasible therapeutic strategies for patients, especially patients with the T315I mutation, is urgently needed. In this study, we explored a new strategy using RAE-1γ enriched CML-specific Dex, which are able to simultaneously prime T lymphocytes and NK cells to induce anti-CML immunity in vivo and in vitro (Fig. 8)*.*

Dex have received widespread attention as potential cell-free vaccines due to their superior antitumor effect versus DC vaccines [52]. First, Dex can spread to tissues efficiently [55]. Second, the shelf life of cryopreserved Dex is longer than that of DCs [56]. Moreover, the capacity of DCs to prime tumor-specific CTLs is weakened after short-term frozen storage [57]. Third, in comparison with DCs, Dex as inert vesicles are more resistant to tumor-induced immunosuppression [52]. Finally, Dex may be more powerful than DCs in priming T lymphocytes and NK cells [27, 56]. Consequently, Dex could have wider applications in cancer research.

The immune response induced by Dex could be influenced by several factors, such as the source of antigens [58], the antigen loading mode of Dex [59] and the maturation status of the source DCs [60, 61]. First, studies have shown that allogeneic whole tumor lysates supply sufficient tumor-associated antigens and are beneficial in eliciting immune responses [29, 62]. In contrast to restricted antigenic peptides or recombinant tumor proteins, whole tumor lysates are human leukocyte antigen (HLA) unrestricted, implying that Dex-based therapy is applicable to all patients without HLA haplotype-related restrictions [58]. Moreover, as the high rate of tumor mutation allows for the loss of target antigens, it is likely that the application of whole tumor lysates would induce a broader and more potent antigen-specific immune response. Additionally, whole tumor lysates from patients have the potential to be used to prepare personalized vaccines [58]. To achieve a satisfactory antitumor immune effect, we chose tumor lysates generated from BP210 cells or imatinib-resistant BP210-T315I cells rather than single peptides as tumor antigens to load onto Dex. Second, the different modes of antigen loading can influence the ability of Dex to stimulate an immune response [59]. There are two methods of antigen loading onto Dex, i.e., direct loading and indirect loading. Direct loading onto Dex is performed under mildly acidic conditions. For indirect loading, DCs are first directly pulsed with antigens, and then Dex, which are loaded with the antigens, are produced. The former method not only has technical difficulties related to in preparation but also is less effective than the latter in vivo [59]. Thus, for the preparation of indirectly antigen-loaded Dex, we first loaded DCs with CML tumor cell lysates to generate CML-specific DCs. Third, according to previous studies, Dex derived from mature DCs have higher levels of immune-associated molecules and exhibit a better antigen-presenting ability than Dex derived from immature DCs [60, 61]. Notably, indirectly loaded Dex derived from mature DCs have indeed been shown to elicit T-cell immune responses in vivo and in vitro [61]. Nevertheless, in those clinical trials, Dex were almost not involved in all the advantages of these factors as described above [17–19, 27]. In our study, to elicit robust and effective antitumor immunity, CML-specific Dex were isolated from the culture medium of CML-specific DCs, which were induced mature with TNF-α.

We noticed that in clinical trials Dex induced less effective antitumor immunity in some patients and could not elicit the anticipated antigen-specific T-cell response. In contrast, NK-cell effector functions were enhanced in most patients after Dex vaccination [17–19, 27]. Several reports have demonstrated that Dex administration induced NK cell activation in NKG2D/NKG2D-L-dependent manner [17, 27]. Furthermore, NKG2D plays a costimulatory role in T cells [33, 34], thus we designed our CML-specific Dex expressing RAE-1γ to augment the cytotoxic and proliferative activities of T cells. Our goal was to generate novel CML-RAE-1γ-Dex that could enhance the anti-CML efficacy of both NK cells and T lymphocytes. In humans, some populations of T cells to bind to the human NKG2D-L MHC class I chain-related protein A (MICA) through both TCR and NKG2D, thus receiving the antigen-specific signals via TCR/MICA and costimulatory signals via NKG2D/MICA [63]. It is known that murine NKG2D binds RAE-1γ in a manner similar to the interaction between human NKG2D and MICA [50], suggesting that RAE-1γ may play an important role in activating T cells in mice. A previous study found that following antigen stimulation, NKG2D/NKG2D-L signaling primes the activity of CTLs after TCR triggering. Importantly, the effects of NKG2D signaling occurred only when the CTLs were MHC matched with the targets [32]. Additionally, some T cells may use NKG2D as a direct activating molecule [64]. In our research, we found that an anti-NKG2D blocking antibody markedly attenuated cytolysis by T cells treated with CML-RAE-1γ-Dex, suggesting that CML-RAE-1γ-Dex overcame poor antigen presentation efficiency and augmented the antitumor T-cell response via an NKG2D-dependent mechanism (Fig. 4b, c). However, Dex not only harbored NKG2D-L, but also expressed BCL2-associated athanogene 6 (BAG6), TNF superfamily ligands (TNF, FasL and TRAIL) and IL-15Rα, which can directly activate NK cells and enhance their cytotoxic activity [52]. This also agrees with our observations, which showed that NKG2D blockade did not completely suppress GzmB production, implying that CML-RAE-1γ-Dex boosted NK-cell function in a partly NKG2D-dependent manner (Fig. 4a). Moreover, previous studies have indicated that IL-15/IL-15Rα signaling possibly contributes to Dex-related NK-cell responses [52, 65, 66]. Further research should be undertaken to investigate whether either the NKG2D/NKG2D-L signaling or IL-15/IL-15Rα signaling plays a more prominent role in CML-RAE-1γ-Dex-mediated NK-cell function. In our study, RAE-1γ-expressing Dex contribute to activate NK cells and T cells. However, the function of tumor-derived extracellular vesicles expressing NKG2D-L is different. These tumor-derived extracellular vesicles downregulate the NKG2D expression on NK cells and T cells and further impair NKG2D-mediated immune response following prolonged exposure, which is an important mechanism of immune escape [67, 68]. Further research is needed to investigate to compare extracellular vesicles from antigen-presenting cells and tumor cells and the optimal incubation time of stimulation.

In this research, we demonstrated that CML-RAE-1γ-Dex displayed remarkable immunostimulatory activity affecting both the innate and adaptive immune systems. However, the clinical effects should be further investigated. As indicated by previous research, NKG2D is expressed on nearly all NK cells, CD8^+^ T cells, γδT cells and a subset of CD4^+^ T cells in humans [30, 69]. This suggests that antigen-loaded Dex decorated with the human NKG2D-L MICA can activate immune cells in CML patients. Although Dex-based therapy has not been reported in CML, CML-RAE-1γ-Dex restored NK-cell and T-cell functions and enhanced antileukemia cytotoxicity in this study. The Dex generated in this study took advantage of multiple immunocyte subpopulations, reducing the possibility of failure caused by limited activation of a single cell subpopulation. Disease resistance to TKIs and relapse caused by minimal residual disease still have not been resolved [70]. Notably, the T315I-mutation is the most frequent mutation and confers resistance to imatinib [4]. To date, no perfect methods for resolving these issues have been explored. In our study, we found that BT-R-Dex treatment inhibited the occurrence and development of CML in mice caused by BP210-T315I cells injection. This method may provide a novel treatment strategy for CML patients with the T315I mutation and is expected to overcome resistance induced by BCR-ABL^T315I^. In addition, both B-R-Dex and BT-R-Dex induced long-term immune memory in mice that underwent tumor cell rechallenge. This result indicated that these Dex might provide durable protection against CML relapse caused by minimal residual disease. Moreover, approximately 25–30% of acute lymphoblastic leukemia (ALL) cases exhibit the characteristic Philadelphia chromosome and positive BCR-ABL expression [71]. Our study may provide an immunotherapeutic method for these diseases with similar characteristics.

Thus, this is the first time that RAE-1γ was used to decorate on Dex pulsed with whole tumor lysates. The novel Dex increased the cytotoxicity of immunocytes and overcame previous limitations related to antigen-presenting characteristics through an NKG2D-dependent mechanism. These CML-RAE-1γ-Dex elicited and augmented NK-cell and CD4^+^ and CD8^+^ T-cell-mediated immune responses and long-lasting immune memory against CML. Moreover, the strong cytotoxicity against CML cells with a resistance mutation was also achieved. This modified Dex-based immunotherapy may represent a promising option for eradicating CML cells.

## Conclusion

In summary, CML-RAE-1γ-Dex were demonstrated to simultaneously activate multiple immune cell types such as NK cells and CD4^+^ and CD8^+^ T lymphocytes, through the NKG2D/NKG2D-L (RAE-1γ) pathway, which led to anti-CML efficacy in vitro and in vivo. The survival time of leukemia-bearing mice was prolonged by CML-RAE-1γ-Dex vaccination, and long-term protective immunity against BCR-ABL and BCR-ABL^T315I^-induced CML was successfully elicited. This approach based on CML-RAE-1γ-Dex vaccines may be a promising strategy for CML treatment, especially for patients with the T315I mutation.

## Supplementary Information

**Additional file 1: Figure S1**. RAE-1γ was expressed in constructed BP210-RAE-1γ and BP210-T315I-RAE-1γ cells and DCs were successfully induced according to the phenotypic properties of DCs. a Parental and transfected CML cells were stained with an anti-RAE-1γ mAb (red line) or isotype control mAb and then assessed by flow cytometric analysis. The black line corresponds to the isotype-matched antibody. b Flow cytometric analysis of CD80, CD86 and MHC class I/II expression on DCs gated on CD11c+ cells before and after cytokine-induced differentiation. The indicated conjugated antibodies are shown in the Materials and Methods.

**Additional file 2: Figure S2**. Effect of CML-RAE-1γ-Dex on NK-cell function. a-c CD69 (a), CD137 (b) and CD107a (c) expression on the surface of NK cells was measured by flow cytometric analysis. Numbers indicate the percentage of cells with positive expression. d-e The functional markers, perforin (d) and GzmB (e) in NK cells were assessed after stimulation with exosomes by intracellular staining and flow cytometric analysis. f CFSE-labeled NK cells were incubated with various stimuli. The percentage of divided cells was evaluated by flow cytometric analysis. g Flow cytometry gating strategy for CD3^−^DX5^+^ cells (NK cells) (right). Isotype control antibodies for the anti-CD3 and anti-DX5 antibodies were also used and are shown (left). h Purified splenic CD3^−^ DX5^+^ cells (NK cells) after MACS sorting. NK cells were identified with an anti-CD3 mAb and anti-DX5 mAb (right). Isotype control mAbs were also used for staining (left).

**Additional file 3: Figure S3**. CML-RAE-1γ-Dex enhanced CD4^+^ T-cell immune responses. a-c The percentages of CD69^+^ (a), CD137^+^ (b) and CD107a^+^ (c) cells in CD4^+^ T subpopulations were detected by flow cytometry. d-e The expression of the cytotoxicity mediators, perforin (d) and GzmB (e) in CD4^+^ T lymphocytes from different exosome treatment groups was examined by intracellular staining and flow cytometric analysis. f Representative flow cytometry plots for CD4^+^ T-cell proliferation. g T cells were classified and identified using the T-lymphocyte biomarkers CD3, CD4 and CD8 (right). Isotype control antibodies for the anti-CD3, anti-CD4 and anti-CD8 are shown on the left.

**Additional file 4: Figure S4**. CML-RAE-1γ-Dex induced CD8^+^ T-cell activation and expansion in vitro. a-c Flow cytometry plots demonstrating CD69^+^ (a), CD137^+^ (b) and CD107a^+^ (c) cells in CD8^+^ T cells from all groups after staining with appropriate primary mAbs. d-e Flow cytometric analysis of perforin (d) and GzmB (e) generated by CD8^+^ T lymphocytes in response to different exosomes. f Representative proliferation of CFSE-labeled CD8^+^ T lymphocytes evaluated by flow cytometric analysis after coculture with the indicated stimulus.

**Additional file 5: Figure S5**. Gross examination of mice in all groups is shown. a Photographs of livers and spleens from mice inoculated with BP210 cells are shown. b Representative livers and spleens from BP210-T315I cell-challenged mice were photographed after being placed in order. c The photographs show differences in the liver and spleen among the groups.

## Data Availability

Not applicable.

## Abbreviations

CML: Chronic myeloid leukemia
Dex: Dendritic cell-derived exosomes
RAE-1γ: Retinoic acid early inducible-1γ
NKG2D: Natural killer group 2 member D
DCs: Dendritic cells
TKI: Tyrosine kinase inhibitor
APCs: Antigen presenting cells
CTL: Cytotoxic T lymphocyte
Th: CD4^+^ T helper
TCR: T cell antigen receptor
MVEs: Multivesicular endosomes
MHC I/MHC II: Major histocompatibility complex class I and II
NK: Natural killer
NKG2D-L: NKG2D ligands
PBS: Phosphate-buffered saline
BMDCs: Bone marrow-derived DCs
mAb: Monoclonal antibody
ELISPOT: Enzyme-linked immunospot assay
ELISA: Enzyme-linked immunosorbent assay
WBC: White blood cell
H&E: Hematoxylin and eosin
FDA: Food and Drug Administration
HLA: Human leukocyte antigen
MICA: MHC class I chain-related protein A
BAG6: BCL2-associated athanogene 6
ALL: Acute lymphoblastic leukemia
