# Supplementary figures and images for: Modified dendritic cell-derived exosomes activate both NK cells and T cells through the NKG2D/NKG2D-L pathway to kill CML cells with or without T315I mutation
Source: Exp Hematol Oncol. 2022 Jun 7;11:36. doi: 10.1186/s40164-022-00289-8 (PMC9172178; doi:10.1186/s40164-022-00289-8)

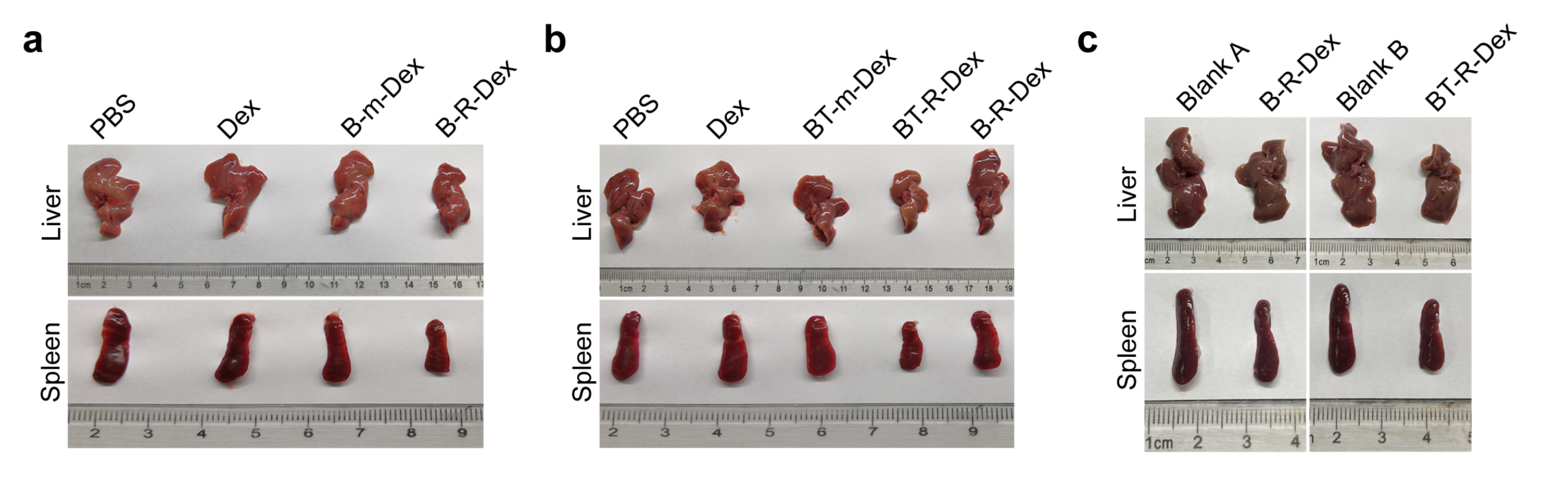

Supplement: Supplementary file 5 — Additional file 5: Figure S5. Gross examination of mice in all groups is shown. a Photographs of livers and spleens from mice inoculated with BP210 cells are shown. b Representative livers and spleens from BP210-T315I cell-challenged mice were photographed after being placed in order. c The photographs show differences in the liver and spleen among the groups. [file 40164_2022_289_MOESM5_ESM.tif]
